# E-cadherin involved in inactivation of WNT/*β*-catenin signalling in urothelial carcinoma and normal urothelial cells

**DOI:** 10.1038/sj.bjc.6601031

**Published:** 2003-06-10

**Authors:** I Thievessen, H-H Seifert, S Swiatkowski, A R Florl, W A Schulz

**Affiliations:** 1 Urologische Klinik, Heinrich-Heine-Universität, Düsseldorf, Germany

**Keywords:** bladder cancer, APC, CDH1, TCF, DNA methylation

## Abstract

Constitutive activation of WNT signalling through *β*-catenin, which leads to increased transcription of TCF/*β*-catenin target genes, is crucial in the development of many human tumour types including colorectal carcinoma and hepatoma. Its role in urothelial cancer (TCC) is unclear, since typical activating mutations are not found. We therefore determined the activity of a *β*-catenin/TCF-dependent promoter in proliferating normal uroepithelial cells and seven TCC cell lines, using a hepatoma line with oncogenic *β*-catenin as a control. Neither normal urothelial cells nor TCC lines exhibited activity under normal growth conditions. In normal cells and 5/7 TCC lines, even transfection of activated *β*-catenin did not restore promoter activity, suggesting repression of *β*-catenin/TCF activity. TCF mRNAs and total *β*-catenin protein levels did not differ qualitatively between inducible and noninducible cell lines, but E-cadherin expression was lacking or low in inducible TCC lines. In these, cotransfection of E-cadherin diminished activation of the TCF-dependent promoter by *β*-catenin. Our results make constitutive WNT/*β*-catenin signalling in TCC appear unlikely, thereby explaining the lack of reported mutations. However, decreased E-cadherin expression occurring in many TCC, often as a consequence of promoter hypermethylation, may confer inappropriate responsiveness to WNT factors.

Inappropriate or autonomous activation of intracellular signal transduction pathways is a major cause for increased proliferation of tumour cells. In several major human cancers, the WNT/*β*-catenin signal transduction pathway is constitutively activated, regularly in colon carcinoma and hepatoma, and with considerable frequency in melanoma, mammary carcinoma, medulloblastoma and in some cases of renal, ovarian, thyroid and prostate carcinomas (Polakis, 2000). In normal tissues, this pathway is crucial for regulation of cell growth and differentiation, from embryonic tissue patterning to adult tissue homeostasis. In particular, WNT signalling may be important for the maintenance of epithelial stem cell compartments (Taipale and Beachy, 2001). The WNT/*β*-catenin pathway elicits activation of specific target genes including the proto-oncogene *c-MYC*, the cell cycle activator *CCND1* (CyclinD1) and the metalloprotease gene *MMP7* (Crawford *et al*, 1999) (see http://www.stanford.edu/~rnusse/wntwindow.html for a list of target genes). In the absence of activating WNT signals, *β*-catenin is phosphorylated by a multiprotein complex containing GSK3*β*, the tumour suppressors APC and axin/conductin. Phosphorylated *β*-catenin is recognised by a ubiquitin–ligase complex with *β*-TrCP as the crucial component, ubiquitinylated and degraded by the proteasome. Binding of an activating WNT factor to the Frizzled receptor leads to the inactivation of GSK3*β* via activation of Dishevelled and GBP/FRAT and thus to stabilisation of *β*-catenin, which accumulates in the cytoplasm, translocates into the nucleus and heterodimerises with TCF/LEF transcription factors to activate target genes containing specific TCF binding sites in their promoters. Constitutive activation of WNT/*β*-catenin signalling in cancer is caused by a variety of mutations in various components of the pathway, leading either to inactivation of APC, axin/conductin or *β*-TrCP or to oncogenic activation of *β*-catenin. In addition, recent evidence suggests that the activity of the pathway may also be influenced by further factors, notably by the status of the E-cadherin cell adhesion protein (Stockinger *et al*, 2001). Whichever mechanism causes constitutive activation of the WNT/*β*-catenin pathway, its activity is reflected in increased transcription of genes containing TCF binding sites.

While the importance of WNT/*β*-catenin signalling is well established in many human carcinomas, its role in transitional cell bladder carcinoma (TCC) is unclear. WNT/*β*-catenin signalling is implicated in urothelial development. Expression of Wnt2b in mesenchymal renal cells induces ureter branching in the murine foetus (Lin *et al*, 2001). In the human foetus, WNT11 is expressed in the tips of the ureter buds and other urogenital tissues (Lako *et al*, 1998). Thus, a function of WNT/*β*-catenin signalling in adult urothelial tissue homeostasis is conceivable. Indeed, differences in WNT7B expression have been reported between normal urothelium, superficial and invasive bladder carcinomas (Bui *et al*, 1998). However, mutations typically responsible for deregulated *β*-catenin/TCF activity in other tumours, that is, in *APC*, *AXIN*, *BTRC* and *CTNNB1*, have not been found in TCC to date (Stoehr *et al*, 2002). This might mean that mutations are rare or absent in this cancer type, that they are difficult to detect, that they occur at unusual sites in the above genes or that they affect different components of the pathway than in other cancers. This is, of course, difficult to ascertain. Two established changes potentially affecting the activity of this pathway in TCC are hypermethylation of the *APC* promoter and diminished expression of E-cadherin, caused by *CDH1* hypermethylation or mutation. These changes have been reported to occur with moderate (*APC*) or high frequency (*CDH1*) in advanced TCC and could conceivably lead to increased *β*-catenin/TCF activity and proliferation in TCC (Maruyama *et al*, 2001; Ribeiro-Filho *et al*, 2002).

To circumvent the vagaries associated with mutation detection in multiple candidate genes and to determine the significance of reported changes in *CDH1* and *APC* in TCC, we have determined WNT/*β*-catenin activity and inducibility in TCC cell lines and in cultured normal uroepithelial cells (NUEC). Transitional cell bladder carcinoma lines harbour genetic changes and gene expression patterns typical of advanced TCC and provide a well-characterised and established experimental system to study properties of this tumour. Normal uroepithelial cells can be maintained in primary cultures, where they proliferate spontaneously or stimulated by external growth factors such as EGF. Furthermore, we analysed the expression of *β*-catenin, APC and E-cadherin at the protein level and expression of TCFs at the mRNA level. The effect of E-cadherin was investigated in more detail. Overall, our data suggest that proliferation of TCC does not depend on activity of the WNT/*β*-catenin pathway, which instead appears to be generally repressed. E-cadherin may act as a buffer for *β*-catenin activity in urothelial cells and its loss in TCC may not only affect cell adhesion, but also sensitivity towards WNT factors.

## MATERIALS AND METHODS

### Cell lines and culture

The bladder carcinoma cell lines, VMCub1, VMCub2, SW1710, SD, HT1376, 5637 and BFTC905, obtained from the DSMZ, Braunschweig, Germany, were cultured in Dulbecco's minimal essential medium (Gibco Life Technologies, Karlsruhe, Germany), supplemented with 15% fetal calf serum and 100 *μ*g/ml penicillin/streptomycin as described (Grimm *et al*, 1995). The hepatoma cell line HepG2, used as positive control because of its oncogenic *β*-catenin mutation, was cultured as described (Schulz *et al*, 1988). Ureters from nephrectomy patients were used to prepare NUEC as described by Southgate *et al* (1994). These were routinely maintained in keratinocyte serum-free medium (KSFM, Gibco Life Technologies) supplemented with 50 *μ*g/ml bovine pituitary extract (BPE), 5 ng/ml epidermal growth factor (EGF) and 30 ng/ml cholera toxin. After the first passage, they were used for experiments as described (Swiatkowski *et al*, 2002).

### Plasmids

The reporter plasmids pTopFlash and pFopFlash, containing wild-type or mutant TCF binding sites, respectively, in front of the HSV thymidine kinase minimal promoter driving the *Photinus pyralis* luciferase gene, were purchased from Upstate Biotechnology (c/o Biomol, Hamburg, Germany). The *Renilla reniformis* luciferase reporter plasmid, pTKRL, used as internal control for transfection efficiencies, and the negative control vector pGL3 were purchased from Promega, Mannheim, Germany. pLINEluc, constructed by insertion of bps −193 to +661 from the active LINE-1 element L1.2B into pGL3 (Steinhoff *et al*, 2002) was used as positive control. The following expression plasmids were used: pbcat, kindly donated by Dr H Clevers, Utrecht, NL, contains human *β*-catenin cDNA with an activating S33Y mutation driven by the strong CMV promoter. pEGFP-UM permits expression of murine E-cadherin cDNA, kindly provided by Dr R Kemler, Freiburg, Germany, from the CMV promoter.

### Transfections and reporter gene assays

Cells were grown in six-well plates to 30% confluence. Transient transfection was carried out using FuGene/DMEM (Roche, Mannheim, Germany) at a 1 : 25 dilution. Per well, 1 *μ*g of reporter plasmid, 0.5 *μ*g of each expression plasmid and 0.15 *μ*g pTKRL were transfected. At 80% confluence, cells were lysed and luciferase activity was measured using the Dual Luciferase Reporter Assay System (Promega, Heidelberg, Germany) as recommended by the manufacturer. Each experiment was repeated with at least five independent passages or cultures.

### Western blotting

Cells were grown in 75 cm^2^ tissue culture flasks to 80% confluence and lysed for 1 h on ice in modified RIPA-buffer (50 mM Tris, pH 7.2, 150 mM NaCl, 40 mM NaF, 5 mM EGTA, 5 mM EDTA, 1 mM sodium orthovanadate, 1% nonidet P-40, 0.1% sodium dodecylsulphate, 0.1% sodium deoxycholate, 10 *μ*g/ml phenylmethylsulphonylfluoride). Protein amounts were quantified by the Bradford method. For SDS–polyacrylamide gel electrophoresis (10% polyacrylamide for *β*-catenin and E-cadherin, 5% for APC), 10 *μ*g of protein were used and subsequently transferred to an Immobilon-P membrane (Millipore Corp., Bedford, MA, USA). After blocking in 10% nonfat milk powder in PBS overnight at 4°C, membranes were incubated for 1 h at room temperature with primary antibodies at the following dilutions: anti-*β*-catenin (BD Transduction Laboratories, Heidelberg, Germany) at 1 : 1500, anti-E-cadherin (Santa Cruz Biotechnology, CA, USA) at 1 : 1000, anti-APC (Upstate Biotechnology, c/o Biomol, Hamburg, Germany) at 1 : 500 and anti-*α*-tubulin (Sigma, St Louis, MO, USA) at 1 : 5000. Incubation with HRP-conjugated rabbit anti-mouse secondary antibody (Santa Cruz Biotechnology) at 1 : 5000 was carried out at room temperature for 1 h, followed by luminescence detection with the ECL-Kit (Amersham-Pharmacia, Freiburg, Germany).

### RNA isolation and RT–PCR

Total mRNA was isolated from cultures grown to 80% confluence, using the RNeasy® Midi Kit (Qiagen, Hilden, Germany). After quantification, mRNA was transcribed into first-strand cDNA using AMV-RT (Promega, Mannheim, Germany). Polymerase chain reactions were carried out in a total 20 *μ*l volume containing 1 × PCR-buffer (Biometra, Göttingen, Germany), 150 *μ*M of each nucleotide, 10 pmol of each primer, 1 U of *Taq* polymerase (Biometra) with 2% (*CTNNB1*), 3% (*TCF1*), 2% (*TCF4*) and 2.5% (*hAES*) formamide added. Each PCR cycle consisted of 30 s denaturing at 95°C, 30 s at the annealing temperature and 1 min at 72°C. Each initial denaturation was performed for 5 min at 95°C and each final extension for 7 min. A total of 25 cycles were performed for *CTNNB1* at 57°C annealing temperature, 34 cycles at 59°C for *TCF1*, 35 cycles at 58°C for *LEF1*, 31 cycles at 59°C for *TCF3*, 29 cycles at 56°C for *TCF4*, and for *hAES*, 36 cycles at 59°C. Primer pairs were selected according to Iwao *et al* (1998) for *CTNNB1*, Duval *et al* (2000) for *TCF4* and Brantjes *et al* (2001) for *TCF1, LEF1, TCF3* and *hAES*. *GAPDH*, with primers added to the mixture for the last 19 cycles, served as an internal control. Polymerase chain reaction products were separated by agarose gel electrophoresis (2%) and visualised by ethidium bromide staining.

### DNA extraction and methylation analysis

High molecular weight genomic DNA from cell lines was isolated using the blood and cell culture DNA kit (Qiagen, Hilden, Germany). MS-PCR was performed essentially as described by Herman *et al* (1996). In short, 1 *μ*g DNA from each cell line was bisulphite-treated with the CpGenome DNA modification kit (Oncor, Heidelberg, Germany). Aliquots from the reaction mixture were then used in two separate PCR amplifications with primer pairs from the *CDH1* and the *APC* gene promoter regions specific for converted, that is unmethylated, or unconverted, that is methylated, DNA using the conditions specified in Herman *et al* (1996) and Virmani *et al* (2001). Polymerase chain reaction conditions were as follows: template DNA (100 ng) was amplified in a total volume of 50 *μ*l containing 150 *μ*M of each dNTP, 1.5 mM MgCl_2_, 15 pmol of each primer, and 1.25 U of HotStar *Taq* polymerase (Qiagen, Hilden, Germany). Following initial denaturation at 94°C for 15 min, 35 cycles of 30 s at 95°C, 30 s at *T*_m_, and 45 s at 72°C were performed. All reactions included a final elongation step at 72°C for 10 min.

## RESULTS

### Properties of cell lines used

Some important properties of the TCC lines used in this study are compiled in Table 1

**Table 1:** Properties of TCC lines

**TCC line**	***TP53*mutation**	**RB Western blot**	***CDKN2A***	***APC*methylation**	***APCWestern blot***	***CDH1*methylation**	**E-Cadherin Western blot**
VmCub1	Mutant	+	Mutant	−	+	−	+
	Exon 5						
VmCub2	Mutant	+	Deleted	−	+	−	−
	Exon 5						
SW1710	Mutant	+	Deleted	−	+	±	−
	Exon 8						
SD	Mutant	+	Deleted	−	+	−	+
	Exon 4						
HT1376	Mutant	−	+	+	+	−	+
	Exon 7						
5637	Mutant	−	+	−	+	−	±
	Exon 8						
BFTC905	Wild-type	+	Deleted	−	+	−	+
	(MDM2↑↑)						

For each cell line, *TP53*, RB and *CDKN2A* status are given as previously described (Steinhoff *et al*, 2002; Swiatkowski *et al*, 2002). *APC* and *CDH1* hypermethylation and expression were determined in the present study as described in the Materials and methods section.

Methylation status: + hypermethylated, − no hypermethylation; ± both hypermethylated and unmethylated alleles detectable; protein expression: + present; − not detectable; ± weakly detectable.

, including *APC* and *CDH1* hypermethylation status, which were newly determined. By MS-PCR, all *APC* alleles were hypermethylated in HT1376, and one allele of *CDH1* was hypermethylated in SW1710.

### Reporter gene analysis of endogenous and inducible *β*-catenin/TCF signalling activity

First, endogenous activity of the WNT/*β*-catenin pathway was determined in proliferating NUEC and seven TCC lines under optimal growth conditions. The hepatoma cell line HepG2 harbouring an activating *β*-catenin mutation was used as a positive control. The cells were transfected with either pTopFlash or pFopFlash plasmids. These plasmids are identical but for mutations in the TCF/*β*-catenin binding sites in the promoter driving luciferase expression. The ratio of luciferase expression from pTopFlash to pFopFlash is an established indicator of Wnt/*β*-catenin pathway activity. Differences in transfection efficiencies were corrected by cotransfection of a *Renilla* luciferase plasmid. Plasmids without a promoter (pGL3) or with a cell-type-independent retrotransposon promoter (pLINEluc) were included in each experiment as quality controls.

Figure 1A

**Figure 1:**
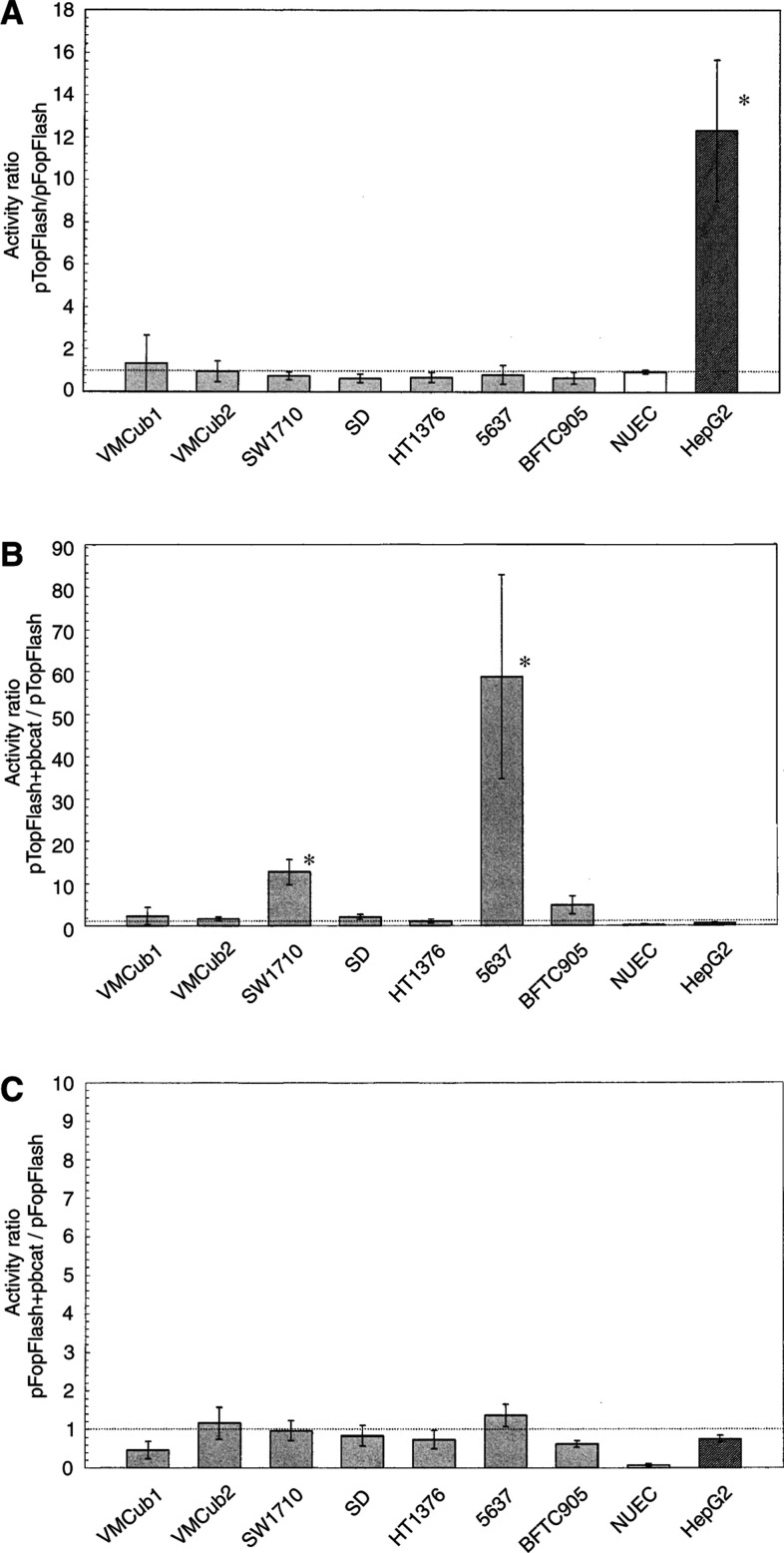
Basal and inducible activities of a TCF/*β*-catenin-dependent promoter in TCC lines and NUEC. The indicated TCC lines, NUEC and the hepatoma cell line HepG2 (hatched bar) were transfected with reporter plasmids and luciferase activity was measured 2 days later. All data are derived from at least five independent triplicate experiments. (**A**) Basal activity of a TCF/*β*-catenin-dependent promoter (contained in pTopFlash) in TCC lines. Mean±s.d. of the pTopFlash/pFopFlash activity ratio are shown. The dotted line indicates a ratio of 1 corresponding to lack of activity. The ratio is significantly different in the HepG2-positive control cell line (^*^t-test; *P*<0.05). (**B**) Induction of pTopFlash reporter activity by oncogenic *β*-catenin. Data are mean±s.d. of the ratio pTopFlash+pbcat/pTopFlash. The dotted line indicates a ratio of 1 indicating lack of inducibility. Statistically significant inducibility (^*^t-test: *P*<0.05) was observed in the TCC lines SW1710 and 5637. (**C**) Effect of oncogenic *β*-catenin on activity of the pFopFlash reporter that contains mutated TCF sites. Data are mean±s.d. of the ratio pFopFlash+pbcat/pFopFlash. The dotted line indicates a ratio of 1 indicating lack of inducibility. No statistically significant inducibility was observed.

shows the pTopFlash to pFopFlash ratio in seven TCC-lines, normal urothelial cells and the positive control HepG2. As expected for a cell line with an activating *β*-catenin mutation, HepG2 displayed significant endogenous signalling activity (12.3±3.3). In contrast, the ratios found in normal urothelial cells (0.96±0.09) and in the TCC lines (range: 0.64±0.20–1.33±1.3) indicate lack of *β*-catenin/TCF signalling.

Next, we tested whether mutationally activated *β*-catenin was able to induce promoter activity from pTopFlash in TCC and NUEC in cotransfection experiments. Figure 1B displays the ratios of luciferase activity with *vs* without *β*-catenin. Surprisingly, in normal urothelial cells and four of seven TCC-lines, the ratio did not differ significantly from 1 with values ranging from 0.2±0.1 in normal urothelial cells to 2.4±2.1 in VMCub1, indicating that expression of activated *β*-catenin was not sufficient to increase transcription from the *β*-catenin/TCF-dependent promoter. In contrast, significant induction was observed in the TCC lines SW1710 (12.7±2.3) and 5637 (58.7±23.8). A slight, but not statistically significant induction was observed in BFTC905 (4.9±2.0). The high basal activity in HepG2 was not further increased by transfected *β*-catenin, as expected. Cotransfection of *β*-catenin did not significantly alter the activity of pFopFlash (Figure 1C) in any cell type.

### Expression of *β*-catenin

**Figure 2:**
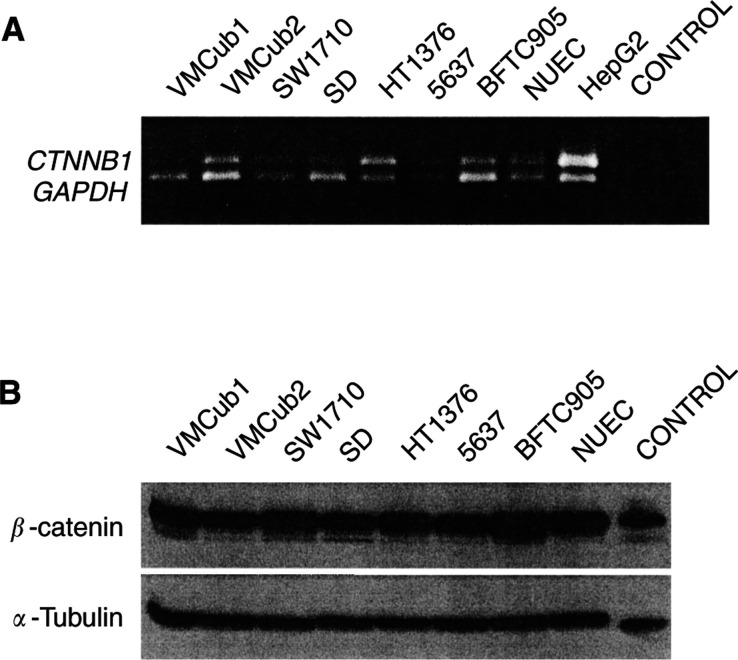
Expression of *β*-catenin in TCC lines and normal uroepithelial cells. Expression of *β*-catenin (*CTNNB1*) was determined in the indicated TCC lines, NUEC and HepG2 hepatoma cells at the mRNA level (**A**) by RT–PCR using GAPDH for comparison and at the protein level (**B**) using *α*-tubulin for comparison. Control in (**A**) refers to PCR without cDNA, the size marker is shown on the right-hand lane. Control in (**B**) was supplied by the antibody manufacturer.

To identify the cause for the different inducibility of the TCC lines by oncogenic *β*-catenin, we first compared the expression of endogenous *β*-catenin. Qualitative RT-PCR analysis revealed *β*-catenin mRNA to be present in all TCC lines and in normal urothelial cells with only slight differences in expression levels (Figure 2A

). Western blot analysis confirmed that overall *β*-catenin expression was also similar in all TCC lines and normal urothelial cells at the protein level (Figure 2B). Thus, the differences in inducibility were not due to major differences in *β*-catenin expression between inducible and noninducible TCC-lines.

### Expression of TCF and hAES mRNA

**Figure 3:**
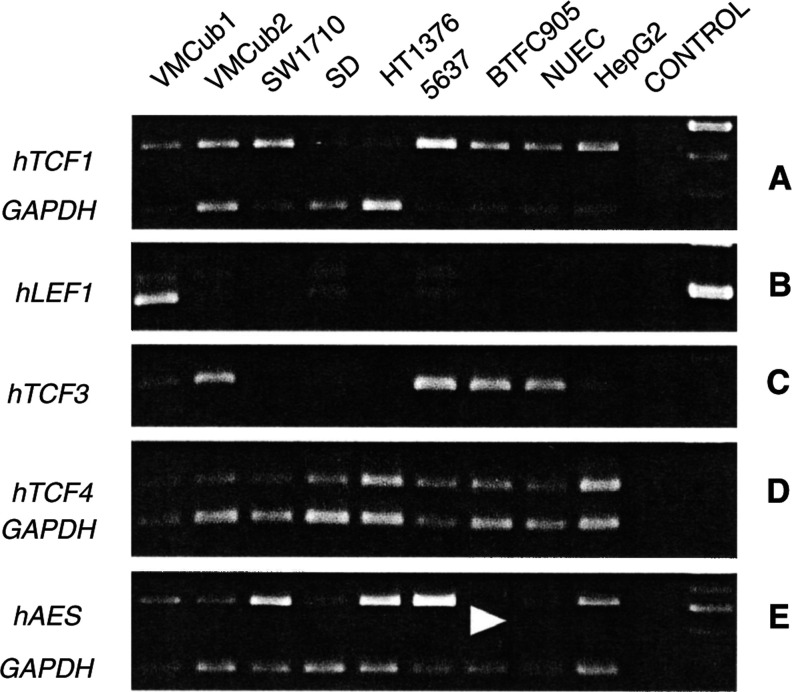
Expression of TCF mRNAs in TCC lines and normal uroepithelial cells. Expression of the indicated TCF mRNAs was determined in the indicated TCC lines, NUEC and HepG2 hepatoma cells by RT–PCR using GAPDH for comparison (except for hLEF1 and hTCF3 yielding similar size PCR products as GAPDH). Control refers to PCR without cDNA, the size marker is shown on the right-hand lane. Note the double band for hLEF1 corresponding to known splice variants and the additional band for hAES in NUEC, which may be a novel splice variant.

*β*-catenin-mediated transcription depends on its interaction in the nucleus with TCF/LEF transcription factors, which in the absence of *β*-catenin act as transcriptional repressors by interacting with transcriptional corepressors of the Grg/TLE-family. Thus, expression patterns of TCF/LEF factors and perhaps of TLE factors could account for the different inducibilities between the TCC lines and were therefore investigated by RT–PCR analysis. *GAPDH* served as internal control. First, we analysed mRNA expression of *hTCF1*, *hLEF1*, *hTCF3* and *hTCF4*. Among these, *hTCF1* and *hTCF4* mRNAs were found in all TCC lines, normal urothelial cells and HepG2 (Figure 3A and D

). Interestingly, in the inducible TCC lines SW1710 and 5637 *hTCF1* expression was slightly, but reproducibly increased relative to *GAPDH* (Figure 3A). Expression of *hLEF1* was more heterogeneous (Figure 3B). The TCC lines SW1710, HT1376 and BFT905, normal urothelial cells and HepG2 displayed only faint bands or lacked *hLEF1* expression, which was robustly detected in VMCub1, VMCub2, SD and 5637. Two bands were found which correspond to known *hLEF1* splice variants (Carlsson *et al*, 1993). While four TCC lines, as well as normal urothelial cells and HepG2 showed *hTCF3* expression, only weak expression could be detected in SD and HT1376, and also a very faint band in the inducible cell line SW1710 (Figure 3C). Neither *hLEF1* nor *hTCF3* expression patterns correlated with inducibility among the TCC lines. Since other Grg/TLE factors have been described as ubiquitous (Brantjes *et al*, 2001), we investigated only *hAES* mRNA by RT–PCR, which is the only member in the Grg/TLE-family that is not a repressor. Figure 3E demonstrates that *hAES* was expressed in all TCC lines, normal urothelial cells and HepG2, except for BFTC905 showing only weak expression. In normal urothelial cells, a second faint band could be observed, which may correspond to a splice variant. As for *hTCF1*, both inducible TCC lines displayed slightly increased *hAES* expression relative to *GAPDH*.

### Role of E-cadherin

**Figure 4:**
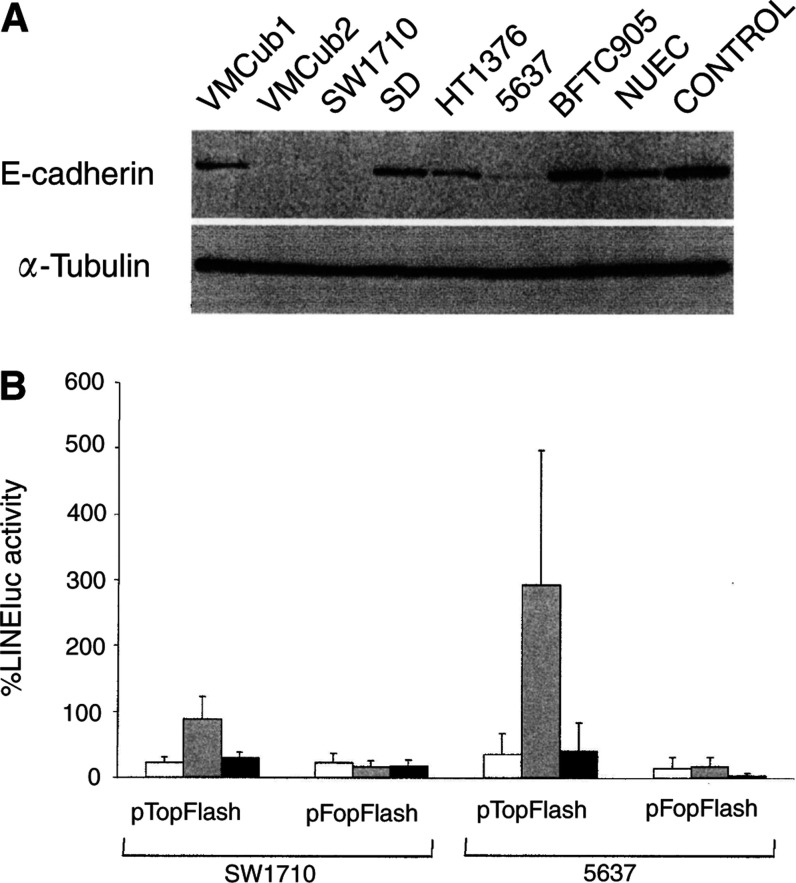
Expression and function of E-cadherin. (**A**) Expression of E-cadherin was determined in the indicated TCC lines and in NUEC by Western blotting (B) using *α*-tubulin for comparison. Control refers to a lysate from cells transfected with an E-cadherin expression construct. (**B**) Effect of E-cadherin cotransfection on induction of pTopFlash reporter activity by oncogenic *β*-catenin in the TCC lines SW1710 and 5637. Values are mean±s.d. of the relative activity of pTopFlash or pFopFlash (as indicated) without any cotransfection (white bars), with transfected pbcat only (grey bars) or pbcat and pEGFP-UM expressing E-cadherin (dark bars). Activity of LINEluc was set as 100%. The differences in pTOP-Flash activation caused by E-cadherin cotransfection are statistically significant (*t*-test: *P*<0.05) in both cell lines.

Since E-cadherin has been reported to act as an inhibitor of *β*-catenin/TCF-mediated transcription by sequestering *β*-catenin at the plasma membrane (Stockinger *et al*, 2001) and loss of E-cadherin to be frequent in TCC (Rieger *et al*, 1995), the differences in WNT/*β*-catenin signalling between the TCC lines could be related to E-cadherin expression. Indeed, the *CDH1* gene encoding E-cadherin was found to be hypermethylated in SW1710 (Table 1). In Western blot analysis, the TCC lines VMCub1, SD, HT1376 and BFTC905 as well as normal urothelial cells were found to express E-cadherin (Figure 4A

). Diminished expression or loss of E-cadherin protein was observed in both inducible TCC lines, SW1710 and 5637, and in the noninducible VMCub2 line. We therefore tested whether re-expression of E-cadherin in the inducible TCC lines SW1710 and 5637 might restore repression of *β*-catenin/TCF signalling in reporter gene analysis. In both cell lines, significant inhibition of promoter activation by *β*-catenin was elicited by E-cadherin cotransfection (Figure 4B). In SW1710 and 5637, induced levels of pTOPluc promoter activity were diminished by 60 and 90%, respectively. Smaller effects were observed on pFOPluc.

### APC expression

In addition to methylation analysis of the *APC* gene, its expression was investigated at the protein level by Western blot analysis. APC protein was present in all TCC lines and normal urothelial cells and even detectable in HepG2 cells, albeit at a lower level. In particular, diminished levels were neither observed in the TCC lines SW1710 and 5637, which were inducible by *β*-catenin, nor in HT1376 cells, which displayed hypermethylation of all *APC* alleles (data not shown).

## DISCUSSION

Constitutive activity of the WNT/*β*-catenin signalling pathway is an important step in the development of many human cancers. However, none of the typical mutations in components of this pathway have been reported in TCC. Few studies have appeared addressing this issue explicitly, which is likely due to publication bias against negative results (Stoehr *et al*, 2002). Still, a lack of mutations in known components of a pathway does not permit the conclusion that it is intact unless its actual functional state has been determined. This is difficult to perform in TCC tissues, but can be done by reporter gene analysis in TCC lines that show the typical genetic aberrations of advanced bladder cancers. The results of the present study are in accord with the impression from the literature that activating mutations in the WNT/*β*-catenin signalling pathway are rare or absent in TCC. Moreover, the data suggest that this pathway is inactive in and not required for proliferation of TCC cells. Since the same result was obtained with proliferating NUEC, the lack of basal activity in TCC cells is likely an extension of the state in the normal tissue. Moreover, even in normal cells, the pathway could not be activated by oncogenically activated *β*-catenin. Thus, the WNT/*β*-catenin signalling pathway is not only inactive, but turned off in urothelial cells, likely because it is not required for proliferation and perhaps to avoid inappropriate target gene activation. More speculatively, it is interesting to consider the downregulation of the WNT/*β*-catenin pathway in urothelial cells and carcinomas in the light of current hypotheses on the function of the pathway in maintaining stem cell properties (Taipale and Beachy, 2001). According to our data, such a function in uroepithelial tissue seems unlikely. This might be related to its particular organisation as a transitional epithelium with its low turnover, in which stem cells may behave differently as in colon.

Nevertheless, the activation of typical WNT/*β*-catenin target genes such as *CCND1* and *MYC* is required for the proliferation of uroepithelial cells, too. Proliferation of NUEC is stimulated by growth factors such as EGF and, in an autocrine fashion, HB-EGF (Freeman *et al*, 1997). Since these factors act via MAPK signalling (Swiatkowski *et al*, 2002), they seem quite sufficient to stimulate the necessary transcription of *CCND1* and *MYC* in an alternative fashion to WNT/*β*-catenin signalling. Almost all advanced TCC display defects in either *RB1* or *CDKN2A*, which obliterate the requirement for cyclin D1 (cf. Table 1). Furthermore, in some cases *CCND1* itself is amplified (Proctor *et al*, 1991). Increased expression of *MYC* is found in almost all TCC, and is correlated with increased gene copy numbers (Christoph *et al*, 1999) and/or overexpression of the EGF receptor (Lipponen, 1995). Thus, upregulation of *MYC* or *CCND1*, required for the proliferation of TCC cells, is likely achieved by mechanisms other than activation of WNT/*β*-catenin signalling.

This interpretation implies that the inducibility of TCF-dependent gene expression found in the TCC lines 5637 and SW1710 is a deviation from the normal state in the urothelium and that at least one of the mechanisms ensuring inactivity of the WNT/*β*-catenin pathway has become defective. We did not observe significant differences between these two cell lines and the others in expression of overall *β*-catenin, *hLEF1*, *hTCF3* and *hTCF4*. However, both cell lines displayed decreased expression of E-cadherin. Indeed, restoration of E-cadherin expression caused repression of TCF/*β*-catenin-induced transcriptional activity (Figure 4B). On a note of caution, our data indicate that E-cadherin expression cannot be the only factor determining inducibility of WNT/*β*-catenin signalling, since VMCub2 cells also lacked the protein, but did not respond to *β*-catenin transfection. Interestingly, both inducible TCC lines, but not VMCub2, displayed slightly increased mRNA expression of *hTCF1* and *hAES*. If hTCF1 acts as a feedback repressor of *β*-catenin/TCF4 signalling (Roose *et al*, 1999), increased hTCF1 expression in SW1710 and 5637 obviously cannot account for their inducibility by *β*-catenin. However, the hAES homologue Grg5 is known to act as a de-repressor of TCF-mediated transcription (Roose *et al*, 1998). Thus, in addition to E-cadherin loss increased hAES levels could contribute to inducibility of *β*-catenin/TCF signalling in SW1710 and 5637. The potential roles of hTCF1 and hAES are more difficult to address experimentally than that of E-cadherin, since the differences in hTCF1 and hAES expression between the cell lines were only quantitative.

The finding that E-cadherin modulates WNT/*β*-catenin signalling in urothelial cells is in line with recent data suggesting this as a function of E-cadherin additional to or coordinate with mediating cell adhesion (Stockinger *et al*, 2001). In this regard, our findings suggest that while constitutive activation by ‘classical’ mutations does not occur, the WNT/*β*-catenin pathway may play a certain role in a subset of TCC, likely those with loss of E-cadherin expression. This subset of tumours may show increased sensitivity towards WNT factors present in the tissue. Indeed, changes in WNT7B expression have been reported in some TCC (Bui *et al*, 1998). Loss of E-cadherin expression in TCC is associated with a worse clinical prognosis (Bornman *et al*, 2001). It might be worthwhile to investigate the emerging connection between WNT expression, E-cadherin loss and clinical prognosis in more detail.

Finally, hypermethylation of the *CDH1* and *APC* genes has been reported in TCC tissues. Hypermethylation of *CDH1* was found to be associated with loss of protein expression, although not all cases with loss of protein also displayed hypermethylation (Bornman *et al*, 2001; Ribeiro-Filho *et al*, 2002). This was reflected in the TCC cell lines investigated here. Downregulation of E-cadherin expression was found in 3/7 cell lines, but hypermethylation in only one. While the significance of CDH1 hypermethylation is thus likely, that of *APC* hypermethylation, reported at frequencies up to 30% (Maruyama *et al*, 2001), is not obvious in TCC. The HT1376 cell line showed methylation of all *APC* alleles, but neither basal activity nor inducibility of the WNT/*β*-catenin pathway were observed. In fact, APC protein expression could be detected in HT1376. While this suggests that APC hypermethylation does not implicate activation of WNT/*β*-catenin signalling, more extensive studies are required on this issue.
